# Association between hospital bed attainment rates and the density of healthcare professionals in Japan: A nationwide ecological study

**DOI:** 10.1016/j.hpopen.2026.100179

**Published:** 2026-07-20

**Authors:** Kazunori Sugimura, Takaomi Kobayashi, Yoshifumi Saijo

**Affiliations:** a Graduate School of Biomedical Engineering, Tohoku University, 6–6-12 Aramaki Aza Aoba Aoba-ku, Sendai, Miyagi 980–8579, Japan; b Takatsu General Hospital, 1–16-7, Kawasaki, Kanagawa 213–0001, Japan; c Education and Research Center for Community Medicine, Faculty of Medicine, Saga University, 5–1-1 Nabeshima, Saga 849–8501, Japan; d Department of Orthopaedic Surgery, Faculty of Medicine, Saga University, 5–1-1 Nabeshima, Saga 849–8501, Japan; e Department of Liberal Arts, Faculty of healthcare and welfare, Saitama Prefectural University, 820 Sannomiya, Koshigaya-shi, Saitama 343–8540, Japan; f Department of Preventive Medicine, Faculty of Medicine, Saga University, 5–1-1 Nabeshima, Saga 849–8501, Japan

**Keywords:** Regional Healthcare Plan, Hospital bed attainment rates

## Abstract

**Background:**

In Japan, the “Regional Healthcare Plan” aims to optimize the allocation of hospital beds based on projected healthcare demand. However, many regions fail to achieve the recommended bed attainment rates, suggesting that factors beyond demand, particularly healthcare workforce supply, may influence regional disparities. This study examined the association between hospital bed attainment rates and the density of multidisciplinary healthcare professionals across Japan.

**Methods:**

We conducted a nationwide cross-sectional ecological study using publicly available data from the 2023 Community Health Care Visions and 2023 Medical Facility Survey. The unit of analysis was the secondary medical field. Hospital bed attainment rates were calculated as the ratio of actual to required beds and were categorized into three groups (<90%, 90–110%, and > 110%) for each bed function (intensive care, acute care, inpatient rehabilitation, and chronic care). The healthcare workforce density (per 100,000 people) was calculated for multiple professions. Group comparisons were performed using the Kruskal–Wallis test with Bonferroni correction, and associations were assessed using Spearman's rank correlation coefficients.

**Results:**

Bed attainment rates varied widely across regions, with only a minority meeting the recommended range, particularly for intensive and acute care beds. The association between bed attainment rates and workforce density differed according to bed function. High total bed attainment rates were associated with a high density of nonphysician professionals, including nurses and rehabilitation staff. Intensive care bed attainment rates were positively associated with physicians and pharmacists. By contrast, acute care bed attainment rates were negatively correlated with physician and pharmacist densities. Inpatient rehabilitation and chronic care bed attainment rates were positively associated with the density of multiple nonphysician professionals, such as therapists, dietitians, and care workers.

**Conclusion:**

The relation between hospital bed attainment rates and healthcare workforce density varies substantially according to bed function. These findings suggest that effective regional healthcare planning should incorporate not only projected demand but also the composition and distribution of the healthcare workforce to avoid mismatches between bed capacity and service delivery.

## Highlights

• Many regions fail to meet target hospital bed attainment rates in Japan.
• Workforce density is linked to bed attainment, varying by bed function.
• Intensive care beds correlate with physician and pharmacist density.
• Nonphysician staff drive rehabilitation and chronic care bed attainment.
• Policy should integrate workforce capacity into bed planning frameworks.

## 1 Introduction

With the rapidly aging population in Japan, healthcare demand for chronic disease management, rehabilitation, and long-term care has increased. Simultaneously, the efficient allocation of healthcare resources has become a critical challenge for ensuring a sustainable healthcare system. In particular, the aging population has led to structural changes in healthcare demand, shifting from acute intensive care to chronic care. Therefore, the appropriate allocation and functional differentiation of hospital beds have become important for organizing an effective healthcare delivery system [1].

The “Japanese Regional Healthcare Plan” was introduced to optimize healthcare resource allocation. Under this framework, future healthcare demand is projected at the secondary medical area level, and the required number of beds is determined for each bed function category [2]. Hospital beds are classified into functional categories: intensive care beds, acute care beds, inpatient rehabilitation beds, and chronic care beds. The ratio of actual to required beds (bed attainment rates) in each region is used as a key policy evaluation indicator [3]. This framework is based on projected healthcare demand, including medium- to long-term changes in population structure as well as the quality and quantity of regional healthcare needs. In the “Japanese Regional Healthcare Plan”, required bed numbers are estimated based on projected healthcare demand. For advanced acute, acute, and recovery care beds, patients are classified according to healthcare resource utilization, measured by reimbursement points under the national fee schedule. Age- and sex-specific inpatient utilization rates are then applied to future population projections to estimate regional healthcare demand. Required bed numbers are calculated by dividing the estimated demand by predetermined occupancy rates (75% for advanced acute care, 78% for acute care, 90% for recovery care, and 92% for chronic care) [2].

However, bed attainment rates have not been achieved in many regions, with a substantial proportion falling outside the 90–110% range. Given the inherent uncertainty in healthcare demand projections, planning targets are typically evaluated within acceptable deviation ranges rather than exact matches 2., [4]. One possible explanation is that this metric does not adequately account for supply-side factors of healthcare delivery, particularly the allocation and composition of multidisciplinary healthcare professionals, including doctors, nurses, therapists, pharmacists, registered dietitians, social workers, and care workers. Therefore, this study aimed to examine the association between bed attainment rates by bed function and the density of multidisciplinary healthcare professionals across secondary medical areas in Japan.

## 2 Materials and methods

### 2.1 Study design

This study was designed as a cross-sectional ecological analysis to examine the association between hospital bed attainment rates and the number of healthcare professionals in Japan. We used data from “the 2023 Community Health Care Visions” [2] and “Overview of the 2023 Medical Facility (Static and Dynamic) Survey and Hospital Report” published by the Ministry of Health, Labour and Welfare [5]. Data from 2023 were used for the analysis because both datasets on hospital bed attainment rates and the density of healthcare professionals were consistently available. The unit of analysis was the secondary medical area, which represents the fundamental geographic unit used for healthcare planning in the Japanese Regional Healthcare Plan.

This study uses publicly available, aggregated, and anonymized data. Individuals with identifiable information were excluded. Therefore, ethics committee approval was not required for this study.

#### 2.1.1 Hospital bed attainment rates

Hospital bed attainment rates were obtained from publicly available datasets related to “the 2023 Community Health Care Visions” published by the Ministry of Health, Labour, and Welfare [2]. Hospital beds were categorized as intensive care beds, acute care beds, inpatient rehabilitation beds, and chronic care beds.

The hospital bed attainment rate (%) for secondary medical areas was defined as (actual number of beds)/(required number of beds) × 100. The secondary medical areas were categorized into three groups according to hospital bed attainment rates: <90%, 90–110%, and > 110%. The ±10% threshold was selected because a 10% change in bed capacity has been used as a policy-relevant benchmark in Japan's hospital bed reorganization program, where financial support is provided to institutions that reduce bed numbers by at least 10%. In addition, given the inherent uncertainty in projections of future healthcare demand, a ± 10% range was considered an appropriate margin for distinguishing substantial shortages or surpluses from regions whose bed supply was broadly consistent with projected demand [6].

#### 2.1.2 Density of healthcare professionals

Data on healthcare professionals were obtained from “Overview of the 2023 Medical Facility (Static and Dynamic) Survey and Hospital Report” published by the Ministry of Health, Labour and Welfare [5]. The healthcare professionals included doctors, nurses, physical therapists, occupational therapists, speech-language pathologists, pharmacists, registered dietitians, social workers, and care workers. To compare urban and rural regions equally, the density of healthcare professionals (per 100,000 people) in each secondary medical area was calculated using the following formula: (number of healthcare professionals)/(population) × 100,000.

### 2.2 Statistical analysis

All analyses were conducted at the regional level. Continuous variables are presented as medians and interquartile ranges because the distribution of healthcare professional density was non-normal. Comparisons across the three attainment groups were performed using the Kruskal–Wallis test. When the overall Kruskal–Wallis test indicated a statistically significant difference, post hoc pairwise comparisons were conducted using the Bonferroni correction to adjust for multiple testing. Statistical significance was set at *p* < 0.05. For post hoc pairwise comparisons among the three groups, a Bonferroni-adjusted significance level of *p* < 0.017 (0.05/3) was applied.

Spearman's rank correlation coefficients were calculated to examine the association between continuous bed attainment rates and healthcare professional density and to assess the robustness of the findings.

All statistical analyses were performed using JMP Pro 17 (SAS Institute, Cary, NC, USA).

## 3 Results

**Fig. 1:**
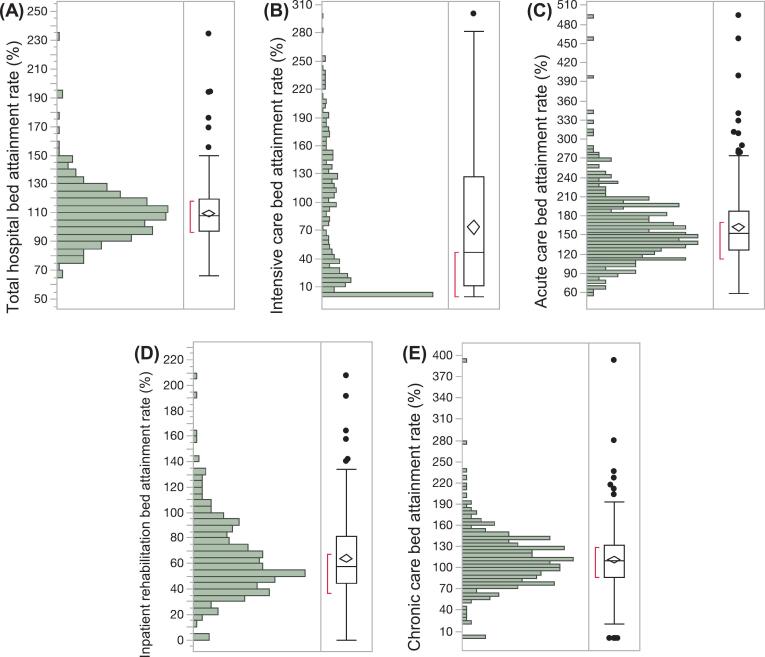
Distribution of bed attainment rates by bed type; (A) Total hospital beds, (B) Intensive care beds, (C) Acute care beds, (D) Inpatient rehabilitation beds, and (E) Chronic care beds. Box-and-whisker plots display the distribution of bed attainment rates. The central line within each box indicates the median, the box represents the interquartile range, and the whiskers indicate the range excluding outliers. Open diamonds denote mean values, and black dots indicate outliers.

Bed attainment rates were widely dispersed across regions, with relatively few areas falling within the recommended 90–110% range for most bed types. Although 41.7% of the total hospital beds were within this range, the corresponding proportions were substantially low for intensive care (7.9%), acute care (8.2%), inpatient rehabilitation (11.2%), and chronic care beds (23.6%; Fig. 1).

The association between the bed attainment rate and healthcare workforce density differed according to bed function. A high total hospital bed attainment rate was associated with an increased density of nonphysician professionals, including nurses and rehabilitation-related staff (Table 1). By contrast, the intensive care bed attainment rate was associated with a high density of doctors and pharmacists (Table 2). The acute care bed attainment rate showed an inverse association with doctor and pharmacist densities (Table 3), whereas the inpatient rehabilitation bed attainment rate was not related to workforce density, except for pharmacists (Table 4). For chronic care beds, a high attainment rate was associated with a low density of multiple nonphysician professionals, whereas doctor density remained unchanged (Table 5).

**Table 1:** Number of healthcare professionals per 100,000 population according to total hospital bed attainment rate (actual/required).

	Total hospital beds (actual/required)			
	<90% (40 areas)	90%–110% (138 areas)	>110% (153 areas)	*p* value
Doctor, *n*	178.7 (136.4, 236.5)	166.9 (133.4, 211.3)	171.9 (144.0, 233.0)	0.485
Nurse, *n*	573.6 (465.9, 674.6)	630.0 (493.0, 764.9)	736.2 (581.3, 896.0)^a^, ^b^	<0.001
Physical therapist, *n*	55.7 (44.4, 83.2)	67.5 (50.5, 88.5)	72.0 (53.3, 92.9)^a^	0.028
Occupational therapist, *n*	30.4 (24.9, 46.2)	36.7 (27.4, 52.3)	45.0 (30.0, 62.1)^a^	0.004
Speech-language pathologist, *n*	12.2 (8.2, 14.7)	12.3 (9.1, 17.2)	13.4 (8.5, 19.1)	0.478
Pharmacist, *n*	37.3 (29.5, 43.2)	36.6 (31.6, 45.2)	38.2 (32.1, 47.3)	0.284
Registered dietitian, *n*	15.0 (12.8, 18.0)	18.1 (13.9, 23.0)^a^	21.0 (15.8, 28.2)^a^, ^b^	<0.001
Social worker, *n*	10.4 (8.7, 13.2)	12.1 (9.4, 15.7)	13.3 (9.8, 18.0)^a^	0.007
Care worker, *n*	24.8 (14.2, 42.2)	26.3 (16.5, 44.0)	35.6 (20.0, 60.2)^a^	0.018

Values are the median (interquartile range) and compared using Kruskal-Wallis test.

a Significantly different (*p* < 0.017) from the values in <90% group using the Bonferroni method.

b Significantly different (*p* < 0.017) from the values in 90%–110% group using the Bonferroni method.

**Table 2:** Number of healthcare professionals per 100,000 population according to intensive care bed attainment rate (actual/required).

	Intensive care beds (actual/required)			
	<90% (197 areas)	90%–110% (26 areas)	>110% (108 areas)	*p* value
Doctor, *n*	159.5 (131.8, 192.4)	179.3 (145.6, 216.7)	205.3 (153.3, 275.2)^a^^b^	<0.001
Nurse, *n*	636.8 (525.0, 775.5)	655.0 (506.4, 870.3)	693.2 (534.6, 852.1)	0.056
Physical therapist, *n*	65.7 (46.0, 90.6)	69.1 (54.3, 93.3)	69.7 (55.8, 87.4)	0.467
Occupational therapist, *n*	39.4 (25.8, 57.3)	36.3 (30.4, 52.1)	38.6 (29.7, 55.8)	0.974
Speech-language pathologist, *n*	11.5 (7.8, 16.7)	13.6 (11.6, 18.3)	14.0 (10.7, 18.9)^a^	0.002
Pharmacist, *n*	34.8 (29.4, 43.2)	42.0 (34.0, 44.8)	43.6 (34.6, 51.5)^a^	<0.001
Registered dietitian, *n*	19.5 (14.5, 25.8)	17.3 (13.9, 25.2)	18.5 (14.8, 23.3)	0.712
Social worker, *n*	12.7 (9.5, 16.7)	11.0 (9.8, 17.0)	12.2 (9.6, 16.0)	0.973
Care worker, *n*	34.1 (16.1, 58.1)	27.4 (15.1, 46.3)	29.2 (18.7, 43.7)	0.352

Values are the median (interquartile range) and compared using Kruskal-Wallis test.

a Significantly different (*p* < 0.017) from the values in <90% group using the Bonferroni method.

b Significantly different (*p* < 0.017) from the values in 90%–110% group using the Bonferroni method.

**Table 3:** Number of healthcare professionals per 100,000 population according to acute care bed attainment rate (actual/required).

	Acute care beds (actual/required)			
	<90% (15 areas)	90%–110% (27 areas)	>110% (289 areas)	*p* value
Doctor, *n*	205.6 (191.7, 275.4)	212.2 (176.7, 289.2)	163.8 (133.7, 208.0)^a^, ^b^	<0.001
Nurse, *n*	671.6 (603.4, 690.8)	710.1 (618.3, 850.0)	649.6 (519.2, 821.2)	0.268
Physical therapist, *n*	56.6 (50.5, 75.7)	74.3 (57.3, 86.8)	66.9 (50.5, 90.1)	0.366
Occupational therapist, *n*	33.2 (25.8, 36.7)	37.9 (32.0, 60.0)	40.3 (27.4, 57.1)	0.186
Speech-language pathologist, *n*	11.7 (8.9, 14.7)	14.0 (11.4, 19.3)	12.4 (8.5, 18.0)	0.414
Pharmacist, *n*	44.6 (33.5, 47.7)	43.5 (39.6, 51.5)	36.4 (31.2, 45.1)^b^	<0.001
Registered dietitian, *n*	18.6 (14.1, 20.7)	19.4 (16.8, 25.2)	19.1 (14.4, 24.9)	0.735
Social worker, *n*	12.3 (9.4, 14.1)	12.3 (10.8, 16.6)	12.5 (9.5, 16.7)	0.793
Care worker, *n*	19.1 (13.8, 34.5)	30.5 (17.1, 40.9)	30.9 (17.5, 53.2)	0.187

Values are the median (interquartile range) and compared using Kruskal-Wallis test.

a Significantly different (*p* < 0.017) from the values in <90% group using the Bonferroni method.

b Significantly different (*p* < 0.017) from the values in 90%–110% group using the Bonferroni method.

**Table 4:** Number of healthcare professionals per 100,000 population according to inpatient rehabilitation bed attainment rate (actual/required).^a^, ^b^

	Inpatient rehabilitation beds (actual/required)			
	<90% (272 areas)	90%–110% (37 areas)	>110% (22 areas)	*p* value
Doctor, *n*	172.8 (139.1, 226.1)	153.4 (132.7, 209.4)	157.2 (132.6, 184.3)	0.246
Nurse, *n*	655.5 (512.6, 825.0)	678.1 (542.3, 801.3)	643.4 (592.1, 824.4)	0.713
Physical therapist, *n*	67.5 (50.7, 88.0)	85.7 (50.0, 118.5)	64.5 (46.2, 81.6)	0.347
Occupational therapist, *n*	38.3 (27.6, 55.3)	49.1 (27.3, 70.0)	37.7 (25.1, 47.8)	0.240
Speech-language pathologist, *n*	13.1 (9.0, 17.8)	11.9 (7.9, 22.1)	10.8 (6.0, 14.2)	0.107
Pharmacist, *n*	38.2 (32.1, 46.7)	34.3 (30.0, 41.0)	34.5 (30.7, 41.8)	0.043
Registered dietitian, *n*	18.6 (14.4, 24.3)	20.7 (15.3, 26.2)	20.7 (15.6, 35.2)	0.240
Social worker, *n*	12.4 (9.6, 16.4)	14.6 (9.3, 18.6)	11.3 (7.0, 18.1)	0.470
Care worker, *n*	29.3 (17.5, 47.6)	36.4 (12.3, 59.4)	33.5 (11.7, 63.7)	0.735

Values are the median (interquartile range) and compared using Kruskal-Wallis test.

a Significantly different (*p* < 0.017) from the values in <90% group using the Bonferroni method.

b Significantly different (*p* < 0.017) from the values in 90%–110% group using the Bonferroni method.

**Table 5:** Number of healthcare professionals per 100,000 population according to chronic care bed attainment rate (actual/required).

	Chronic care beds (actual/required)			
	<90% (91 areas)	90%–110% (78 areas)	>110% (162 areas)	*p* value
Doctor, *n*	162.1 (128.5, 202.8)	182 (141.7, 232.7)	172.7 (143.7, 233.5)	0.059
Nurse, *n*	579.4 (491.3, 674.1)	667.0 (527.2, 828.6)^a^	704.7 (570.0, 873.6)^a^	<0.001
Physical therapist, *n*	55.7 (43.1, 75.7)	69.8 (54.7, 88.3)^a^	72.2 (56.5, 93.5)^a^	<0.001
Occupational therapist, *n*	31.1 (23.8, 46.5)	40.3 (29.3, 57.3)^a^	43.9 (31.2, 62.0)^a^	<0.001
Speech-language pathologist, *n*	10.7 (7.7, 15.4)	13.0 (10.7, 18.5)^a^	14.0 (9.6, 18.4)^a^	0.002
Pharmacist, *n*	34.5 (28.5, 41.6)	39.5 (33.7, 46.6)^a^	38.7 (32.3, 47.4)^a^	<0.001
Registered dietitian, *n*	15.5 (12.5, 20.8)	19.0 (14.2, 23.7)^a^	20.7 (16.1, 28.1)^a^, ^b^	<0.001
Social worker, *n*	10.1 (7.8, 13.6)	13.3 (10.5, 16.4)^a^	13.2 (9.8, 18.1)^a^	<0.001
Care worker, *n*	23.2 (11.6, 43.5)	29.9 (17.5, 48.5)^a^	34.2 (20.4, 56.1)^a^	0.003

Values are the median (interquartile range) and compared using Kruskal-Wallis test.

a Significantly different (*p* < 0.017) from the values in <90% group using the Bonferroni method.

b Significantly different (*p* < 0.017) from the values in 90%–110% group using the Bonferroni method.

Table 6 shows Spearman's rank correlation coefficients to examine associations between continuous bed attainment rates and healthcare professional density. For total hospital bed attainment rates, positive correlations were observed with nurse density (*ρ* = 0.263), occupational therapists (*ρ* = 0.149), registered dietitians (*ρ* = 0.305), social workers (*ρ* = 0.142), and care workers (*ρ* = 0.129). For intensive care bed attainment rates, doctor density (*ρ* = 0.396), nurse density (*ρ* = 0.182), speech-language pathologists (*ρ* = 0.242), and pharmacist density (*ρ* = 0.384) showed positive correlations. Acute care bed attainment rates were negatively correlated with doctor density (*ρ* = −0.298), speech-language pathologist density (*ρ* = −0.170), and pharmacist density (*ρ* = −0.238). For inpatient rehabilitation bed attainment rates, positive correlations were observed with nurse density (*ρ* = 0.264), physical therapists (*ρ* = 0.300), occupational therapists (*ρ* = 0.338), speech-language pathologists (*ρ* = 0.199), registered dietitians (*ρ* = 0.328), social workers (*ρ* = 0.272), and care workers (*ρ* = 0.272). For chronic care bed attainment rates, positive correlations were observed with nurse density (*ρ* = 0.246), physical therapists (*ρ* = 0.187), occupational therapists (*ρ* = 0.178), speech-language pathologists (*ρ* = 0.156), pharmacists (*ρ* = 0.149), registered dietitians (*ρ* = 0.318), social workers (*ρ* = 0.177), and care workers (*ρ* = 0.181).

**Table 6:** Spearman's rank correlation coefficients to examine associations between continuous bed attainment rates and healthcare professional density.

	Total hospital beds (actual/required)	Intensive care beds (actual/required)	Acute care beds (actual/required)	Inpatient rehabilitation beds (actual/required)	Chronic care beds (actual/required)
Doctor	−0.020	0.396****	−0.298****	0.017	0.102
Nurse	0.263****	0.182****	−0.060	0.264****	0.246****
Physical therapist	0.105	0.085	−0.103	0.300****	0.187****
Occupational therapist	0.149**	0.039	−0.030	0.338****	0.178**
Speech-language pathologist	0.020	0.242****	−0.170***	0.199****	0.156***
Pharmacist	0.039	0.384****	−0.238****	0.055	0.149**
Registered dietitian	0.305****	−0.034	0.036	0.328****	0.318****
Social worker	0.142**	0.005	−0.001	0.272****	0.177**
Care worker	0.129*	−0.059	0.044	0.182****	0.181****

*p < 0.05, **p < 0.01, ***p < 0.005, ****p < 0.001.

## 4 Discussion

This nationwide ecological study found that the association between hospital bed attainment rates and the density of healthcare professionals differed according to bed function. Our main findings were as follows: (1) the intensive care bed attainment rate was associated with high densities of doctors and pharmacists, (2) acute care bed attainment rate showed an inverse association with doctor and pharmacist densities, (3) inpatient rehabilitation bed attainment rate was correlated with the densities of nonphysician professionals, and (4) chronic care bed attainment rate was associated with increased densities of nonphysician professionals. These findings suggest that effective regional healthcare planning should consider the composition of the healthcare workforce in addition to bed allocation.

In the present study, the intensive care bed attainment rate was associated with a high density of doctors and pharmacists. Similarly, the delivery of intensive care depends on the availability of intensivist doctors and well-educated staff, which are associated with low mortality and improved clinical outcomes in critically ill patients [7], [8], [9]. Moreover, intensive care units rely on dedicated multidisciplinary teams, including pharmacists, as core components for safe and effective care delivery [10], [11].

We observed that the acute care bed attainment rate showed an inverse association with doctor and pharmacist densities. This finding may be explained by structural factors within the Japanese healthcare system. To improve access to medical care during the postwar period, particularly following the establishment of universal health insurance in 1961, Japan expanded hospital bed capacity, many of which were classified as acute care beds regardless of their actual clinical function [12], [13]. Although acute care beds increased nationwide, doctors and pharmacists subsequently concentrated in urban areas because of better professional opportunities and living conditions, leading to a shortage of these professionals in rural regions [14]. However, the mismatch between acute care beds and professionals in rural regions persists because, according to Japan's healthcare system, maintaining acute care bed capacity can be an important strategy for stabilizing institutional finances. Indeed, according to Roemer's law [15], “a built bed is a filled bed,” suggesting that acute care bed capacity itself can generate demand for hospital services despite low doctor and pharmacist densities.

In this study, inpatient rehabilitation bed attainment rates were correlated with the density of nonphysician professionals. In inpatient rehabilitation beds, patient outcomes (including functional recovery, independence in activities of daily living, and discharge outcomes), particularly among older adults and patients with complex medical conditions, depend on the coordination of multiple professionals (including physical therapists, occupational therapists, speech–language pathologists, dietitians, and medical social workers), rather than advanced medical treatment by doctors [16], [17], [18], [19]. Therefore, regions with many nonphysician healthcare professionals may be better able to expand and operate inpatient rehabilitation beds efficiently.

We found that the chronic care bed attainment rate was associated with increased densities of nonphysician professionals but not with doctor density. Similar to our findings, prior studies on long-term care systems emphasized that care quality and safety in chronic care beds depend primarily on the availability, stability, and skill mix of nursing and care workers rather than on doctor supply alone [20], [21], [22]. These results indicate that the care needs of chronically ill and functionally dependent populations include continuous nursing care, rehabilitation support, nutritional management, and social assistance rather than advanced medical treatment.

These findings have important implications for regional healthcare planning and bed reallocation policies. In Japan, regional healthcare plans have primarily focused on adjusting the number of beds by aligning them with projected healthcare demands (including medium- to long-term changes in population structure and the quality and quantity of regional healthcare needs) [1], [13]. However, our results suggest that healthcare professionals influence not only projected healthcare demand but also current healthcare supply. Bed redistribution without parallel consideration of healthcare workforce composition may result in structural mismatches between nominal capacity and actual service delivery. For example, expanding inpatient rehabilitation or chronic care beds without securing sufficient numbers of nonphysician professionals may compromise the quality of care and operational sustainability [16], [20]. While doctor supply is critical for intensive care delivery, rehabilitation- and chronic-care-oriented systems depend more heavily on multidisciplinary, nonphysician professionals.

The strengths of this study include the use of comprehensive nationwide claims data covering more than 95% of healthcare services in Japan, enabling a detailed regional-level analysis of bed function and workforce distribution [23]. However, this study had some limitations. First, and most importantly, the estimated bed demand used in this analysis was derived from projections developed by the Ministry of Health, Labour and Welfare. Therefore, the validity of our findings depends on the accuracy of these underlying estimates. In addition, the estimation method assumes that age-specific hospitalization rates, healthcare utilization patterns, case mix, and occupancy rates will remain relatively stable over time. It also assumes continued reductions in the average length of hospital stay, although future changes in clinical practice and patient characteristics may differ from these projections. Furthermore, the official projection framework applies standardized occupancy assumptions according to bed function categories and does not explicitly account for potential variation in occupancy rates according to hospital size [24]. Future changes in medical practice, healthcare policy, demographic trends, or population health needs may affect the accuracy of projected bed demand. In particular, age-specific hospitalization rates may change over time rather than remain constant, and future reductions in the average length of stay may not be fully realized in clinical practice. Previous studies have suggested that healthcare utilization is more strongly associated with proximity to death than with chronological age alone. Consequently, regions experiencing greater increases in mortality may have higher hospital bed demand than predicted by models based primarily on age structure [25]. This perspective is not explicitly incorporated into the current projection framework, which primarily relies on demographic projections and age-specific healthcare utilization patterns [26], [27], [28]. Therefore, these factors should be considered when interpreting the results of this study. Second, this was a cross-sectional study; thus, the causal relation between hospital bed attainment rates and the density of healthcare professionals remains unclear. Furthermore, unmeasured factors, such as hospital ownership and local policy initiatives, may have influenced our results. Future longitudinal studies are needed to evaluate temporal changes in bed attainment rates and healthcare workforce distribution and to better clarify potential causal relationships. Third, bed attainment rates are aggregated regional indicators and may not fully capture hospital-level staffing strategies or efficiency.

## 5 Conclusions

In this nationwide ecological study, the association between hospital bed attainment rates and healthcare professional density varied according to bed function. These findings suggest that effective regional healthcare planning should consider the composition of healthcare professionals in addition to hospital bed allocation.

## CRediT authorship contribution statement

**Kazunori Sugimura:** Conceptualization. **Takaomi Kobayashi:** Formal analysis, Data curation. **Yoshifumi Saijo:** Investigation.

## Ethical approval

Not applicable.

## Patient consent for publication

Not applicable.

## Funding

None.

## Declaration of competing interest

The authors declare that they have no known competing financial interests or personal relationships that could have appeared to influence the work reported in this paper.
